# Supplementary material for: Prediction of Microvascular Invasion in Hepatocellular Carcinoma via Deep Learning: A Multi-Center and Prospective Validation Study
Source: Cancers (Basel). 2021 May 14;13(10):2368. doi: 10.3390/cancers13102368 (PMC8156235; doi:10.3390/cancers13102368)
Supplement: Supplementary file 1 [file cancers-13-02368-s001.zip › supplementary materials - xml/Supplementary File S1-S4.pdf]

**File S1. Histopathological diagnosis of microvascular invasion (MVI)**

MVI was defined as the presence of tumor thrombi within small peritumoral vessels (branches of portal vein, hepatic vein, or a large capsular vessel of the surrounding hepatic tissue lined by endothelium) detected only on microscopy [1, 2].

To ensure adequate and reliable detection of MVI, a 7-site sampling procedure was performed at each center. All the HCC specimens were cut apart along the maximal tumor section and were then sliced into serial 1-cm thick sections parallel to the maximal tumor section, with the total number of sections determined by the tumor size. Afterwards, one piece of tissue was each sampled at the transition area between tumor and surrounding liver tissues with a ratio of 1:1 at 12, 3, 6 and 9 o'clock from the less bleeding and necrotic sections. One piece was sampled from the tumor area free from bleeding and necrosis, and one piece each was sampled respectively from proximal (≤1.0 cm to the tumor) and distant liver parenchyma (> 1 cm away from the tumor). Thus, at least 7 tissue blocks were sampled. These tissue blocks were first sampled crossing the maximal tumor section. If the number of tissue block acquired from this section could not reach more than 7, the remaining tissue blocks were sampled from sections elsewhere of the specimens.

Two experienced pathologists independently reviewed all specimen slices for each tissue block to determine the presence of MVI at each site. If MVI was detected in any one of all tissue clocks, MVI was reported as positive for this patient.


[1] Roayaie S, Blume IN, Thung SN, et al. A system of classifying microvascular invasion to predict outcome after resection in patients with hepatocellular carcinoma. Gastroenterology 2009;137(3):850-855.

[2] Rodriguez-Peralvarez M, Luong TV, Andreana L, et al. A systematic review of microvascular invasion in hepatocellular carcinoma: diagnostic and prognostic variability. Ann Surg Oncol 2013;20:325-339.

**File S2: Detailed imaging acquisition protocols**

**1. Magnetic resonance (MR) protocols**

Gadoxetic acid-enhanced MR imaging (EOB-MRI) was performed using various MR scanners (see below in the Table entitled "**MR units used in the study**"). The routine MR sequences included: i) breath-hold fat-suppressed fast spin-echo or turbo spin-echo T2-weighted imaging; ii) in- and opposed-phase gradient-echo or fast spin-echo T1-weighted sequence, iii) diffusion-weighted sequence (b values: West China Hospital and Henan Provincial People's Hospital-0, 50, 500, 800, 1000, and 1200s/mm$^2$ ; Zhongshan Hospital-0, 500 s/mm$^2$), and vi) a fat-suppressed three-dimensional (3D) gradient-echo T1 weighted sequence before and after intravenous injection of gadoxetic acid (EOB) at the arterial phase ([AP] bolus triggering, 20-30s), portal venous phase ([PVP] 60-70s), transitional phase (3 min) and hepatobiliary phase ([HBP], 20min). A standard dose (0.025 mmol/kg) of EOB (Primovist®; Bayer Schering Pharma AG, Berlin, Germany) was injected at a rate of 1.0-2.0 ml/s, with an immediately followed 20-30 ml saline flush through an antecubital venous catheter with a dual power injector.

**MR units used in the study.**

| Hospital | MR unit | Coil |
|---|---|---|
| West China Hospital, Sichuan University | 3.0T Siemens Magnetom Skyra | 18-channel body array coil |
| Henan Provincial People's Hospital | 3.0T GE Discovery MR 750 | 8-channel body array coil |
| Zhongshan Hospital, Fudan University and Shanghai Institute of Medical Imaging | 1.5T Siemens Magnetom Skyra | 8-channel body array coil |

**Detailed imaging parameters of EOB-MRI.**

| Sequence | MR unit | Fat suppression | TR (ms) | TE (ms) | Flip angle (°) | ST (mm) | Matrix | FOV (mm$^2$) |
|---|---|---|---|---|---|---|---|---|
| T2-weighted imaging | 3.0T Siemens Magnetom Skyra | Used | 2160 | 100 | 160 | 6 | 320×288 | 433×433 |
| | 3.0T GE Discovery MR 750 | Used | 6315 | 78 | 15 | 7 | 288×244 | 360×280 |
| | 1.5T Siemens Magnetom Skyra | Used | 4918 | 106 | 160 | 5.5 | 384×273 | 285×380 |
| Dynamic T1-weighted imaging | 3.0T Siemens Magnetom Skyra | Used | 3.95 | 1.92 | 9 | 2 | 352×256 | 400×296 |
| | 3.0T GE Discovery MR 750 | Used | 4.1 | 1.9 | 15 | 5 | 512×512 | 380× 300 |
| | 1.5T Siemens Magnetom Skyra | Used | 3.47 | 1.36 | 10 | 3 | 320×240 | 308×380 |
| T1-weighted in- and opposed-phase imaging | 3.0T Siemens Magnetom Skyra | Not used | 81 | 1.4 | 70 | 6 | 352×286 | 400×325 |
| | 3.0T GE Discovery MR 750 | Not used | 180 | 2.1 | 80 | 6 | 384×160 | 360× 360 |
| | 1.5T Siemens Magnetom Skyra | Used | 6.88 | 2.39/4.77 | 10 | 3.5 | 320×240 | 356×380 |
| Diffusion-weighted imaging | 3.0T Siemens Magnetom Skyra | Used | 5600 | 68 | 90 | 6 | 100×76 | 380×289 |
| | 3.0T GE Discovery MR 750 | Used | 9230 | Minimum | 15 | 5 | 128 × 128 | 360× 380 |
| | 1.5T Siemens Magnetom Skyra | Not used | 5100 | 55 | 90 | 5.5 | 192×154 | 285×380 |

TR=repetition time; TE=echo time; ST=section thickness; FOV=field of view.

**2. Computed tomography (CT) protocols**

Multiphasic contrast-enhanced CT (CE-CT) was performed using various multi-detector CT scanners (see below in the Table entitled "**CT units used in the study**"). All patients received a nonionic contrast medium (Iopamidol, 370 mg of iodine per milliliter [Iopamidol 370; Bracco Imaging, Milan, Italy]) at a dose of 1.4 mL (518 mg of iodine) per kilogram of body weight at a rate of 3.0-4.0 ml/s. Pre-warmed contrast material was administered intravenously at a rate of 2-2.5 ml/s through an 18-gauge intravenous catheter inserted into an antecubital vein.

After acquisition of an anteroposterior digital scout radiograph, patients were scanned craniocaudally from the dome of the liver to the iliac crest before and after intravenous contrast medium administration. Images were obtained during the AP (bolus tracking, 15-20 s after trigger thresholds ranging between 170-182 Hounsfield units [HU] were reached in the supra-coeliac abdominal aorta), PVP (70-80s after the administration of contrast media), and equilibrium phase (180s after the administration of contrast media).

**CT units used in the study.**

| Hospital | CT unit |
|---|---|
| West China Hospital, Sichuan University | Siemens Somatom Definition AS<br>Siemens Somatom Definition Flash<br>GE Revolution CT |
| The First Affiliated Hospital of China Medical University | Siemens Somatom Definition Flash<br>GE Revolution CT |
| Beijing Youan Hospital, Capital Medical University | GE LightSpeed |

**Detailed imaging parameters of multiphasic CE-CT.**

| | Definition AS | Definition Flash | Revolution | LightSpeed |
|---|---|---|---|---|
| No. of channels | 64 | 128 | 256 | 64 |
| Tube voltage (kV) | 120 | 120 | 120 | 120 |
| Tube current (mA) | 420 | 420-520 | 450 | 380 |
| Rotation period (s) | 0.5 | 0.5 | 0.5 | 0.6 |
| Detector collimation (mm) | 76.8 | 76.8 | 80 | 40 |
| Helical pitch | 0.6 | 0.6-1.0 | 0.992 | 0.984 |
| Acquisition time (s) | 4-8 | 2-8 | 2-6 | 4-8 |
| Section thickness (mm) | 2-5 | 1.5-8 | 1.25-5 | 5 |
| Intersection gap | 0 | 0 | 0 | 0 |
| Reconstruction kernel | soft tissue | soft tissue | standard | standard |
| Volumetric CT dose (mGy) | 14.06 | 14.17-17.57 | 14.17-17.57 | 17.13 |

**File S3. Definitions regarding radiological indicators for microvascular invasion (MVI) and radiological confirmation of high-risk MVI areas**

**1. Radiological indicators for MVI on gadoxetic acid-enhanced magnetic resonance imaging (EOB-MRI)**

1) ***Tumor size:*** largest outer-edge-to-outer-edge dimension of the tumor, including "capsule" in measurement [1].
2) ***Multifocality (solitary vs. multiple)*:** number of definite intrahepatic HCC lesions with characteristic enhancement pattern [2, 3].
3) ***Tumor margin (smooth vs. non-smooth)*:** smooth tumor margin was defined as nodular tumors with smooth contours in all imaging planes; whereas non-smooth tumor margin was defined as non-nodular tumors with irregular margin and budding portion at the tumor periphery in the transverse and/or coronal imaging planes [4, 5].
4) ***Tumor capsule (absent vs present)*:** existence of a smooth, uniform, sharp border around most or all of the tumor, unequivocally thicker or more conspicuous than fibrotic tissue around background nodules [1, 5].
5) ***Enhancement pattern (typical vs atypical):*** typical enhancement pattern of HCC was defined as arterial phase hyperenhancement and washout on the portal venous phase [1, 3]; whereas atypical enhancement pattern of HCC was defined as enhancement patterns other than the typical pattern described above.
6) ***Peritumoral enhancement (absent vs present)*:** existence of a detectable portion enhancing in the arterial phase, adjacent to the tumor border, later becoming isointense as compared with the background liver parenchyma [4, 5].
7) ***Hypointensity of the tumor in the hepatobiliary phase (HBP) (absent vs present)*:** signal intensity of the tumor on HBP images unequivocally less, in whole or in part, than liver [1, 5].
8) ***HBP peritumoral hypointensity (absent vs present)*:** existence of wedge-shaped or flame-like hypointense area adjacent to the tumor border on HBP [5].

**2. Radiological indicators for MVI on contrast-enhanced computed tomography (CE-CT)**

1) ***Tumor size:*** largest outer-edge-to-outer-edge dimension of the tumor, including "capsule" in measurement [1].
2) ***Multifocality (solitary vs. multiple)*:** number of definite intrahepatic HCC lesions with characteristic enhancement pattern [2, 3].
3) ***Tumor margin (smooth vs. non-smooth)*:** smooth tumor margin was defined as nodular tumors with smooth contours in all imaging planes; whereas non-smooth tumor margin was defined as nonnodular tumors with irregular margin and budding portion at the tumor periphery in the transverse and/or coronal imaging planes [4, 5].
4) ***Tumor capsule (absent vs present)*:** existence of a smooth, uniform, sharp border around most or all of the tumor, unequivocally thicker or more conspicuous than fibrotic tissue around background nodules [1].
5) ***Enhancement pattern (typical vs atypical):*** typical enhancement pattern of HCC was defined as arterial phase hyperenhancement and washout on the portal venous phase or equilibrium phase [1-3]; whereas atypical enhancement pattern of HCC was defined as enhancement patterns other than the typical pattern described above.
6) ***Peritumoral enhancement (absent vs present)*:** existence of a detectable portion enhancing in the arterial phase, adjacent to the tumor border, later becoming isoattenuating as compared with the background liver parenchyma [4].


[1] CT/MRI LI-RADS version 2018. American College of Radiology. https://www.acr.org/Clinical-Resources/Reporting-and-Data-Systems/LI-RADS/CT-MRI-LI-RADS-v2018. Accessed October 17, 2019.

[2] European Association for the Study of the Liver. EASL Clinical Practice Guidelines: Management of hepatocellular carcinoma. J Hepatol 2018;69(1):182-236.

[3] Omata M, Cheng AL, Kokudo N, et al. Asia-Pacific clinical practice guidelines on the management of hepatocellular carcinoma: a 2017 update. Hepatol Int 2017;11(4):317-370.

[4] Renzulli M, Brocchi S, Cucchetti A, et al. Can Current Preoperative Imaging Be Used to Detect Microvascular Invasion of Hepatocellular Carcinoma? Radiology 2016;279(2):432-442.

[5] Lee S, Kim SH, Lee JE, et al. Preoperative gadoxetic acid-enhanced MRI for predicting microvascular invasion in patients with single hepatocellular carcinoma. J Hepatol 2017;67(3):526-534.

**File S4. Detailed architecture and construction process of the CDLMs**

The primary branch of the developed DL model was ResNet18 which was pretrained on a public dataset – ImageNet and was fine-tuned using our collected retrospective cohorts. We chose the front 13 layers of ResNet18 including 6 residual blocks as the main network for our model training. Besides, a global average pooling layer and softmax layer were added to extract the hidden feature information and calculate the probability of MVI, respectively. The architecture of ResNet18 branch was shown in **Figure S1**.

During training, the gradient of the binary cross-entropy loss was computed on each batch training samples using backpropagation algorithm, parameters were updated in the direction opposite the gradient by SGD algorithm. Hyper-parameters including batch size, learning rate, and weight were adjusted to ensure the optimized prediction performance. The batch size was fixed to 64. The other two hyper-parameters varied on different single EOB-MRI sequence and CE-CT phase, the best learning rate on arterial phase, portal vein phase, delayed phase, T2-weighted imaging, hepatobiliary phase T1-weighted imaging were 0.01, 0.01, 0.001, 0.01, 0.01, while the optimized sample weights between positive and negative cases were 1.0, 0.8, 1.2, 1.0, 1.5 for EOB-MRI, and for CE-CT, the best learning rate on plain phase, arterial phase and portal vein phase were 0.01, the best sample weights were 1.8, 0.8, and 1.4 respectively.

After the branch network of each sequence/phase was well-trained completely, fusion deep learning model of EOB-MRI and CE-CT were developed by adding multiple fully connected layers before the probability output layer (**Figure S2**). Specifically, we froze layers before the global average pooling layer of each sub-branch, then concatenated the output of the assigned layer, and 1280 nodes. Thus, 1280 (256×5) features on EOB-MRI and 768 (256×3) features on CE-CT were obtained as the input of fully connected layer. Three fully connected layers and a softmax layer were finally determined. The numbers of nodes in these three layers were 1024, 512 and 128. The optimization process was the same as described above. The learning rate for EOB-MRI and CE-CT were separately 0.01 and 0.001. The sample weight was 1.0.

Moreover, to validate the value of clinical and radiological characteristics, effective clinical and radiological risk factors were added to deep learning feature nodes (1285 nodes on EOB-MRI and 772 nodes on CE-CT) for Combined Deep Learning Models (CDLMs). The learning rate of combined model on CT and MR were 0.1 and 0.01 respectively. The sample weight was 1.0. The input images contained ROIs of the largest tumor section and its two neighboring sections of each patient. Thus, 987 training and 345 test images were obtained for DL model building and evaluation. The tumor images were standardized by z-score to alleviate the effect brought by image intensity variance among different equipment in different centers. Then, all the images were resized to 64×64 by third-order spline interpolation. Data augmentation was performed to extend our samples and reduce the overfitting by random crop, rotation and flip, etc. All the process of DL model building and evaluation were implemented using the pytorch v1.0.1 deep-learning library on a machine with an NVIDIA GeForce GTX 1080Ti GPU.
