# Supplementary material for: Prediction of Microvascular Invasion in Hepatocellular Carcinoma via Deep Learning: A Multi-Center and Prospective Validation Study
Source: Cancers (Basel). 2021 May 14;13(10):2368. doi: 10.3390/cancers13102368 (PMC8156235; doi:10.3390/cancers13102368)
Supplement: Supplementary file 1 [file cancers-13-02368-s001.zip › supplementary materials - xml/Supplementary Table 1.pdf]

Table 1. Stratification analysis CDLM$^{EOB-MRI}$ and CDLM$^{CE-CT.}$

Different models		CDLM^{CE-CT} Retrospective training cohort (n=306) SEN	SPE	ACC	AUC (95% CI)	Prospective validation cohort 2 (n=115) SEN	SPE	ACC	AUC (95% CI)
**Age (years)**	≤50	68.8%	94.7%	82.8%	0.850 (0.755-0.944)	48.5%	88.9%	66.7%	0.668 (0.528-0.808)
	>50	44.3%	95.2%	80.1%	0.834 (0.778-0.890)	57.1%	91.2%	78.2%	0.825 (0.780-0.941)
	***p* value**	0.037	1		0.786	0.733	1		0.091
**Gender**	Male	64.9%	86.2%	79.2%	0.826 (0.770-0.882)	61.5%	86.3%	75.6%	0.747 (0.642-0.852)
	Female	72.0%	95.6%	87.1%	0.888 (0.804-0.972)	53.3%	70.0%	60.0%	0.687 (0.466-0.907)
	***p* value**	0.684	0.143		0.232	0.81	0.421		0.631
**α-fetopro-tein**	<20ng/mL (A)	56.4%	92.3%	83.3%	0.797 (0.712-0.882)	76.5%	56.5%	65.0%	0.660 (0.480-0.840)
	20–400 ng/mL (B)	90.6%	65.9%	76.3%	0.859 (0.779-0.939)	75.0%	68.2%	70.6%	0.735 (0.552-0.918)
	>400 ng/mL (C)	80.7%	86.1%	83.8%	0.881 (0.801-961)	80.0%	68.8%	75.6%	0.703 (0.524-0.881)
	***p* value**	A *vs* B: 0.010 A *vs* C: 0.177 B *vs* C: 1	A *vs* B: <0.001 A *vs* C: 1 B *vs* C: 0.156		A *vs* B: 0.887 A *vs* C: 0.484 B *vs* C: 1	A *vs* B: 1 A *vs* C: 0.456 B *vs* C: 1	A *vs* B: 1 A *vs* C: 0.313 B *vs* C: 1		A *vs* B: 1 A *vs* C: 1 B *vs* C: 1
**Tumor mar-gin**	Smooth margin	95.0%	88.6%	91.4%	0.951 (0.920-0.982)	73.0%	83.3%	77.1%	0.804 (0.687-0.921)
	Non-smooth mar-gin	88.2%	95.5%	93.8%	0.971 (0.942-0.999)	57.4%	90.0%	79.1%	0.845 (0.771-0.920)
	***p* value**	0.372	0.106		0.361	0.245	0.611		0.563
**Peritumoral enhance-ment**	Present	77.4%	89.2%	83.9%	0.878 (0.815-0.941)	53.3%	76.5%	61.7%	0.586 (0.411-0.761)
	Absent	51.0%	93.5%	82.5%	0.803 (0.734-0.873)	50.0%	95.5%	79.4%	0.758 (0.632-0.883)
	***p* value**	0.03	1		0.36	1	0.239		0.368
	Atypical	84.0%	69.2%	72.4%	0.802	66.7%	80.0%	75.0%	0.6

					(0.716-0.889)				(0.094-1)
**Enhancement Pattern**	Typical	77.9%	82.3%	80.5%	0.862 (0.808-0.917)	60.8%	85.7%	73.8%	0.748 (0.654-0.842)
	***p* value**	1	0.130		0.754	1	1		
**Tumor size**	2cm-5cm	88.0%	57.1%	64.2%	0.784 (0.692-0.875)	53.3%	74.1%	66.7%	0.603 (0.424-0.781)
	>5cm	98.1%	81.1%	89.6%	0.962 (0.931-0.992)	71.8%	76.9%	73.9%	0.712 (0.577-0.847)
	***p* value**	0.541	0.020		0.001	1	1		0.433

CDLM$^{EOB-MRI}$

**Different models**		**Retrospective training cohort (n=329)**				**Prospective validation cohort 2 (n=115)**			
		**SEN**	**SPE**	**ACC**	**AUC (95% CI)**	**SEN**	**SPE**	**ACC**	**AUC (95% CI)**
**Age (years)**	≤50	100%	91.4%	94.5%	0.968 (0.938-0.999)	78.8%	66.7%	73.3%	0.750 (0.621-0.880)
	>50	89.6%	91.7%	91.0%	0.959 (0.934-0.985)	76.2%	76.5%	76.4%	0.869 (771-0.967)
	***p* value**	0.101	1		0.650	1	0.575		0.155
**Gender**	Male	95.9%	91.5%	93.0%	0.966 (0.946-0.986)	71.8%	84.3%	78.9%	0.836 (753-0.918)
	Female	94.1%	74.1%	81.8%	0.928 (0.847-1)	46.7%	90.0%	64.0%	0.753 (0.543-0.964)
	***p* value**	1	0.016		0.376	0.157	1		0.481
**α-fetoprotein**	<20ng/mL (A)	93.3%	94.6%	94.3%	0.973 (0.952-0.994)	88.2%	60.9%	72.5%	0.792 (0.642-0.941)
	20–400 ng/mL (B)	87.0%	94.9%	91.4%	0.948 (0.900-0.996)	91.7%	54.6%	67.7%	0.765 (0.605-0.926)
	>400 ng/mL (C)	95.7%	81.5%	88.0%	0.971 (0.935-1)	88.2%	60.9%	72.5%	0.804 (0.641-0.967)
	***p* value**	A *vs* B: 1<br>A *vs* C: 1<br>B *vs* C: 1	A *vs* B: 1<br>A *vs* C: 0.163<br>B *vs* C: 0.127		A *vs* B: 1<br>A *vs* C: 1<br>B *vs* C: 1	A *vs* B: 1<br>A *vs* C: 0.924<br>B *vs* C: 1	A *vs* B: 1<br>A *vs* C: 0.405<br>B *vs* C: 1		A *vs* B: 1<br>A *vs* C: 1<br>B *vs* C: 1
**Tumor margin**	Smooth margin	95.0%	88.6%	91.4%	0.951 (0.920-0.982)	72.4%	79.0%	75.0%	0.762 (0.619-0.905)
	Non-smooth margin	88.2%	95.5%	93.8%	0.971 (0.942-0.999)	52.0%	90.5%	76.1%	0.837 (0.741-0.933)
	***p* value**	0.372	0.106		0.361	0.205	0.409		0.397

**Peritumoral enhance-ment**	Present	96.2%	84.4%	90.8%	0.931 (0.877-0.986)	78.1%	78.3%	78.2%	0.807 (0.682-0.932)
	Absent	93.4%	92.4%	92.6%	0.968 (0.946-0.991)	27.3%	94.7%	70.0%	0.776 (0.653-0.900)
	***p* value**	0.808	0.182		0.219	<0.001	0.123		0.733
**Tumor size**	2cm-5cm	95.1%	94.6%	94.8%	0.973 (0.950-0.995)	80.0%	63.0%	69.1%	0.704 (0.537-0.871)
	>5cm	100.0%	11.1%	78.4%	0.895 (0.775-1)	100.0%	26.9%	70.8%	0.788 (0.665-0.911)
	***p* value**	0.575	<0.001		0.217	0.027	0.018		0.428

SEN, sensitivity; SPE, specificity; ACC, accuracy; AUC, area under the curve; 95% CI, 95% confidence interval, EOB-MRI, gadoxetic acid-enhanced magnetic resonance imaging; CE-CT, contrast-enhanced computational tomography; T2WI, T2-weighted imaging; HBP, hepatobiliary phase; T1WI, T1-weighed imaging; CDLM, combined deep learning model.
