# Supplementary figures and images for: Prediction of Microvascular Invasion in Hepatocellular Carcinoma via Deep Learning: A Multi-Center and Prospective Validation Study
Source: Cancers (Basel). 2021 May 14;13(10):2368. doi: 10.3390/cancers13102368 (PMC8156235; doi:10.3390/cancers13102368)

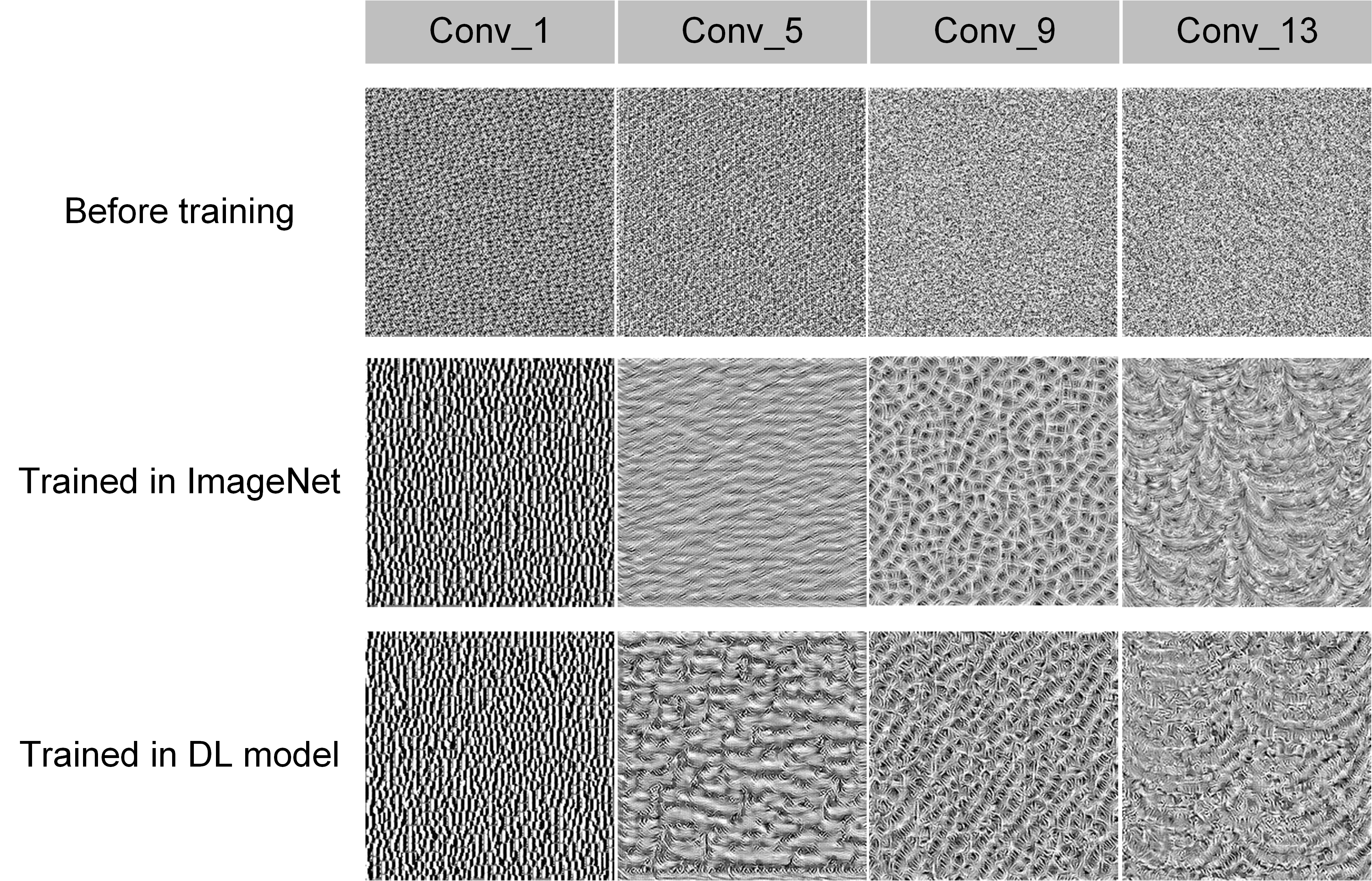

Supplement: Supplementary file 1 [file cancers-13-02368-s001.zip › supplementary materials - xml/Supplementary Figure S3.tif]
